# Acteoside as a potential therapeutic option for primary hepatocellular carcinoma: a preclinical study

**DOI:** 10.1186/s12885-020-07447-3

**Published:** 2020-09-29

**Authors:** Di Ma, Juan Wang, Lu Liu, Meiqi Chen, Zhiyong Wang

**Affiliations:** 1 Department of General Surgery, Ruijin Hospital, Shanghai Jiaotong University School of Medicine, Shanghai, 200025 China; 2 China State Key Laboratory of New Drug & Pharmaceutical Process, Center for Pharmacological Evaluation and Research, Shanghai Institute of Pharmaceutical Industry, 1111 Rd. Zhongshanbeiyi, Hongkou, Shanghai, 200437 China

**Keywords:** Acteoside, Hepatocellular carcinoma, Sorafenib, Xenograft, p53, Kallikrein

## Abstract

**Background:**

Hepatocellular carcinoma (HCC) is a common malignant tumor with characteristics of poor prognosis, high morbidity and mortality worldwide. In particular, only a few systemic treatment options are available for advanced HCC patients, and include sorafenib and the recently described atezolizumab plus bevacizumab regimen as possible first-line treatments. We here propose acteoside, a phenylethanoid glycoside widely distributed in many medicinal plants as a potential candidate against advanced HCC.

**Methods:**

Cell proliferation, colony formation and migration were analyzed in the three human HCC cell lines BEL7404, HLF and JHH-7. Angiogenesis assay was performed using HUVESs. The BEL7404 or JHH-7 xenograft nude mice model was established to analyze the possible antitumor effects of acteoside. qRT-PCR and western blotting were used to reveal the potential antitumor mechanisms of acteoside.

**Results:**

Acteoside inhibited cell proliferation, colony formation and migration in all the three human HCC cell lines BEL7404, HLF and JHH-7. The prohibition of angiogenesis by acteoside was revealed by the inhibition of tube formation and cell migration of HUVECs. The combination of acteoside and sorafenib produced stronger inhibition of cell colony formation and migration of the HCC cells as well as of angiogenesis of HUVECs. The in vivo antitumor efficacy of acteoside was further demonstrated in BEL7404 or JHH-7 xenograft nude mice model, with an enhancement when combined with sorafenib in inhibiting the growth of JHH-7 xenograft. Further treatment of JHH-7 cells with acteoside revealed an increase in the level of tumor suppressor protein p53 as well as a decrease of kallikrein-related peptidase (KLK1, 2, 4, 9 and 10) gene level with no significant changes of the rest of KLK1–15 genes.

**Conclusions:**

Acteoside exerts an antitumor effect possibly through its up-regulation of p53 levels as well as inhibition of KLK expression and angiogenesis. Acteoside could be useful as an adjunct in the treatment of advanced HCC in the clinic.

## Background

Hepatocellular carcinoma (HCC) is a common malignant tumor with characteristics of poor prognosis and high morbidity and mortality worldwide [1, 2]. Various factors have been identified to contribute to the occurrence and progression of HCC, including the infection by hepatitis B or C virus, the inactivation of tumor suppressor genes such as p53, the abnormal activation of oncogenes such as K-ras and some signaling molecules such as PI3K, ERK/MAPK and Wnt/β-catenin as well as the evasion of the host immune system [3, 4]. For the management of HCC, resection, liver transplantation and radiofrequency ablation are options for early-stage HCC patients, yet with high rates of recurrence and metastasis [3, 5, 6]. In addition, only a few systemic treatment options are available for advanced HCC patients, and include sorafenib and the recently described atezolizumab plus bevacizumab regimen as possible first-line treatments [7]. In this context, it is of importance and interest to either develop new agents targeting multiple molecular targets or set up a new strategy such as application of combined therapy in the treatment of advanced HCC.

Acteoside is a phenylethanoid glycoside widely distributed in many medicinal plants, including *Ligustrum purpurascens* [8], *Rehmannia glutinosa* [9] and *Ligstrum purpurascens* [10]. Mounting evidence has shown that acteoside can exert various biological activities, including its antitumor activity. For example, acteoside showed strong antiproliferative effects on prostatic cancer cells [11]. Intraperitoneal delivery of acteoside suppressed tumor growth in a melanoma mouse model possibly through inhibiting protein kinase C [12]. In addition, acteoside potentiated the sensitization of colorectal cancer cells to 5-Fluorouracil via targeting PI3K/Akt signaling [13]. Our previous data have also shown an inhibition of melanogenesis by acteoside in B16 melanoma cells [14]. Despite that, little information is available regarding the possible efficacy of acteoside in the treatment of HCC and the potential underlying mechanisms.

In the present study, we have shown that acteoside inhibits cell proliferation, colony formation and migration in all the three human HCC cell lines BEL7404, HLF and JHH7. The angiogenesis is also inhibited following treatment with acteoside, as shown by the inhibition of tube formation and cell migration of HUVECs. The in vivo antitumor efficacy of acteoside is further demonstrated in BEL7404 or JHH-7 xenograft nude mice model, with an enhancement when combined with sorafenib in inhibiting the growth of JHH-7 xenograft. The antitumor effects of acteoside could be ascribed to its up-regulation of p53 as well as suppression of KLKs and angiogenesis.

## Methods

### Cell culture

The human hepatocellular carcinoma (HCC) cell lines BEL7404, HLF and JHH7 were obtained from the American Type Culture Collection and were routinely cultured in DMEM medium containing 10% fetal bovine serum supplemented with 100 units/ml penicillin G and 100 μg/ml streptomycin in a humidified incubator at 37 °C with 5% carbon dioxide. Human umbilical vein endothelial cells (HUVECs) were isolated from the human umbilical cord veins (provided by ScienCell Research Laboratories, San Diego, USA) and maintained in an endothelial cell medium as described previously [15].

### Reagents

Acteoside (A01) was purchased from Jiangsu Yongjian Medicine Technology Co., Ltd. (Taizhou, China; Lot No. 100581; Purity≥99% by HPLC). Sorafenib was obtained from Selleck Chemicals (Houston, USA). We dissolved acteoside in 0.9% saline. The stock solution of sorafenib was prepared in dimethyl sulfoxide (DMSO; 10 mM), aliquotted and stored at − 20 °C.

### Animals

Male BALB/c (Nu/Nu) mice (18-20 g, 6–8 weeks at the time of receipt) were used in the study (Shanghai Slac Laboratory Animal Co. Ltd., Shanghai, China). Animals were housed in individually ventilated polysulfone cages in a temperature- and light-controlled room (12 h light-12 h dark cycle, lights on at 7:00 a.m.) with free access to food and water. All experimental protocols were approved by the Institutional Animal Care and Use Committee and performed in accordance with the guidelines of the International Association for the Study of Pain concerning the use of laboratory animals.

### Cell proliferation assay

The anti-proliferative activity was performed using the MTT assay. Briefly, cells were plated at a density of 4–5 × 10^3^ cells per well in 96-well plates. After attached overnight, cells were treated with a series of concentrations of acteoside. Following the drug treatments at the indicated time point, cells were incubated with 3-(4,5-Dimethylthiazol-2-yl)-2,5-diphenyltetrazolium bromide (MTT; Roche Diagnostic Corporation, Indianapolis, IN, USA. Final concentration:5 μg/ml) for 3–4 h at 37 °C. The formazan product was dissolved in DMSO for quantitation at a wavelength of 570 nm.

### Colony formation assay

Cells were seeded in 24-well plates at a density of 200 cells per well and allowed to attach to the bottom of the well overnight. Cells were then treated with indicated drugs at the given concentration. The culture medium was replaced every 3 days until colonies were visible at day 12 post-culturing. For colonies counting, the cells were fixed with 4% paraformaldehyde and stained with 0.1% crystal violet. Colonies> 10 cells were counted under a light microscope (× 100 magnification; Olympus, Tokyo, Japan).

### Wound healing assay

The cells were seeded in 6-well plates (1 × 10^5^ cells per well) and cultured to 90% confluency. The cell layer was scratched with a sterile 200 μl pipette tip to produce a wound gap. Cell migration into the wounded area was visualized at indicated time points (0, 10 and 24 h) under an inverted light microscope at a magnification of × 100. Each experiment was performed in triplicate.

### Tube formation assay

The HUVECs were cultured as previously described [15]. In brief, the 96-well plates were coated with Matrigel (BD Biosciences, Franklin Lakes, USA) according to the manufacturer’s instructions and then returned to the incubator to polymerize for 30–40 min. The HUVECs were then seeded at a density of 2 × 10^5^ cells/mL and treated with various given drugs for 8 h. Tube formation was photographed with an inverted microscope at the indicated time points and analyzed using an ImageJ software.

### Western blotting analysis

Total cell lysates were prepared with radio-immunoprecipitation assay buffer (RIPA) (50 mM Tris-HCl at pH 8.0, 150 mM NaCl, 1% NP40, 1% sodium deoxycholate, 0.1% SDS, 10 μg/ml leupeptin, 10 μg/ml aprotinin and 2 mM PMSF). Protein samples with equal amounts (20 μg/lane) were separated on SDS-PAGE gel and transferred to PVDF membranes (Bio-Rad, Hercules, CA). The membranes were incubated overnight with p53 antibody (Cell Signaling Technology, Boston, MA). For loading controls, the membranes were rinsed with a stripping buffer and reprobed with the β-actin antibody (Santa Cruz Biotechnology, Santa Cruz, CA). Proteins were detected using an ECL detection system (Perkin Elmer Life Sciences, Boston, USA).

### qRT-PCR

Total RNA was extracted from cell lines using the Trizol reagent (Invitrogen, USA) and underwent a reverse transcription-polymerase chain reaction (RT-PCR) to synthesize cDNA using a commercially available kit. The real-time qRT-PCR using 2xSYBR green qPCR Supermix was conducted on the Stratagene Mx3000P PRC machine (Agilent Technologies, USA). The primer sequences were shown below: sense 5′-CACCATGTGGTTCCTGGTTC-3′ and anti-sense 5′- CAAACAAGTTGTGGCGACCC-3′ for KLK1; sense 5′-GTGTACAGTCATGGATGGGC-3′ and anti-sense 5′-CCCAGAATCACCCCCACAAG-3′ for KLK2; sense 5′- CCACACCCGCTCTACGATATGA-3′ and anti-sense 5′-CCCAGAATCACCCGAGCAG-3′ for KLK3; sense 5′-CGCACACTGTTTCCAGAACTC-3′ and anti-sense 5′- GTTGCAGGAGTCCTTCTGGT-3′ for KLK4; sense 5′-CCTGCACCCACATCTTTCTCT-3′ and anti-sense 5′-GGTAAGCATCCTCGCACCTT-3′ for KLK5; sense 5′- CTCTCTCCTGGGGACACAGA-3′ and anti-sense 5′-TCCGCCATGCACCAACTTAT-3′ for KLK6; sense 5′-TTTTGGAGCCCAGCTGTGTG-3′ and anti-sense 5′-GTCACCATTGCAGGCGTTTT-3′ for KLK7; sense 5′-CTGGGCAGGACACTCCAG-3′ and anti-sense 5′-ACACCGCCACAGAGTAGTTG-3′ for KLK8; sense 5′-GTAGGGGGTTCTCGTAGGGT-3′ and anti-sense 5′-CGGTGACGTCATAGAGACGG-3′ for KLK9; sense 5′-CAGGAGTGCCAGCCTCAC-3′ and anti-sense 5′- CTGGGGAGGAAGAGGATGGA-3′ for KLK10; sense 5′-CCCACCCCTTGGATTCTGTCT-3′ and anti-sense 5′-GTGAACTATGTAGCGGGGCT-3′ for KLK11; sense 5′-TGTTCTTGGTGAGTTCTCCCG-3′ and anti-sense 5′-GGATCCAGTCCACATACTTGC-3′ for KLK12; sense 5′-CCTGAACCACGACCATGACA-3′ and anti-sense 5′-GCAGGGTTTGGATGTAGCCT-3′ for KLK13; sense 5′-CCCAACTACAACTCCCGGAC-3′ and anti-sense 5′-CCTGAAGCAACTGCTCGTGA-3′ for KLK14; sense 5′-AGTTGCTGGAAGGTGACGAG-3′ and anti-sense 5′-TGGCTAACATCTGGGCCTTG-3′ for KLK15; sense 5′-GACAGTCAGCCGCATCTTCT-3′ and anti-sense 5′- GCGCCCAATACGACCAAATC-3′ for GAPDH. The cycle threshold (Ct) values of each KLK gene were normalized to that of GAPDH and analyzed with the MxPro software.

### In vivo antitumor activity of A01 alone or in combination with sorafenib

The BEL7404 cell suspensions were prepared (3 × 10^6^ cells) and injected subcutaneously into the right flank of athymic nude mice. Ten days after the inoculation, tumor-bearing mice (body weight(g):22–26; mean ± SEM:23.8 ± 0.14) were screened and selected for randomization into 7 treatment groups in accordance with tumor volume (70-120 mm^3^). A total of 38 mice were assigned to different treatment groups (*n* = 5 per group except for the model group (*n* = 8)). Animals were then treated via oral gavage with acteoside (A01; 12.5, 25 and 50 mg/kg), sorafenib alone (50 mg/kg) or in combination with A01 (50 mg/kg) once daily for 14 days. Moreover, a dose of 20 mg/kg A01 was also delivered intravenously. Doses were selected based on previous studies and our preliminary data [15]. The tumor growth was monitored every 3 or 4 days using an electronic caliper, with the volume calculated as 0.5xlengthxwidth^2^ (mm^3^). In addition, a parallel experiment using JHH-7 inoculation was included. Seven days after the inoculation, tumor-bearing mice (body weight(g):20–26; mean ± SEM:24.0 ± 0.20) were screened and selected for randomization into treatment groups in accordance with tumor volume (80-130 mm^3^). A total of 23 mice were assigned to 4 treatment groups (*n* = 5 per group except for the model group (*n* = 8)). Animals then received A01 (20 mg/kg, iv), sorafenib alone (50 mg/kg, ig) or combined with A01 (20 mg/kg, iv) once daily for 14 consecutive days. All mice were anesthetized with chloral hydrate (350 mg/kg) and sacrificed by neck dislocation at the end of the observation period. And tumor mass was removed and pictured with a digital camera.

### Statistical analysis

All data are expressed as mean ± standard deviation (SD). Unless otherwise stated, statistical analyses were performed using analysis of variance (ANOVA) followed by a *post-hoc* Tukey test. Tumor volume values are shown as mean ± standard error of mean (SEM). Two-way ANOVA followed by Tukey tests were used to analyze tumor volume data. The GraphPad Prism software was used for statistical analyses. A value of *p* < 0.05 was considered statistically significant.

## Results

### Effects of A01 on the proliferation and colony formation of HCCs in vitro

**Fig. 1:**
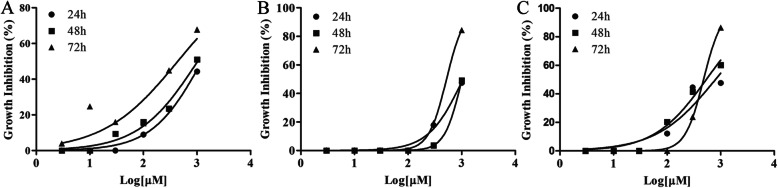
Acteoside inhibits cell proliferation in HCC cells. The three human HCC cell lines BEL7404 (**a**), HLF (**b**) and JHH (**c**) were treated with different doses of acteoside (3, 10, 30, 100, 300 and 1000 μM) following an overnight attachment in 96-well plates. Cell proliferation was detected with MTT at 24 h, 48 h and 72 h post-treatment. Each treatment was performed in triplicate. The dose-response curve was fitted with nonlinear regression

**Fig. 2:**
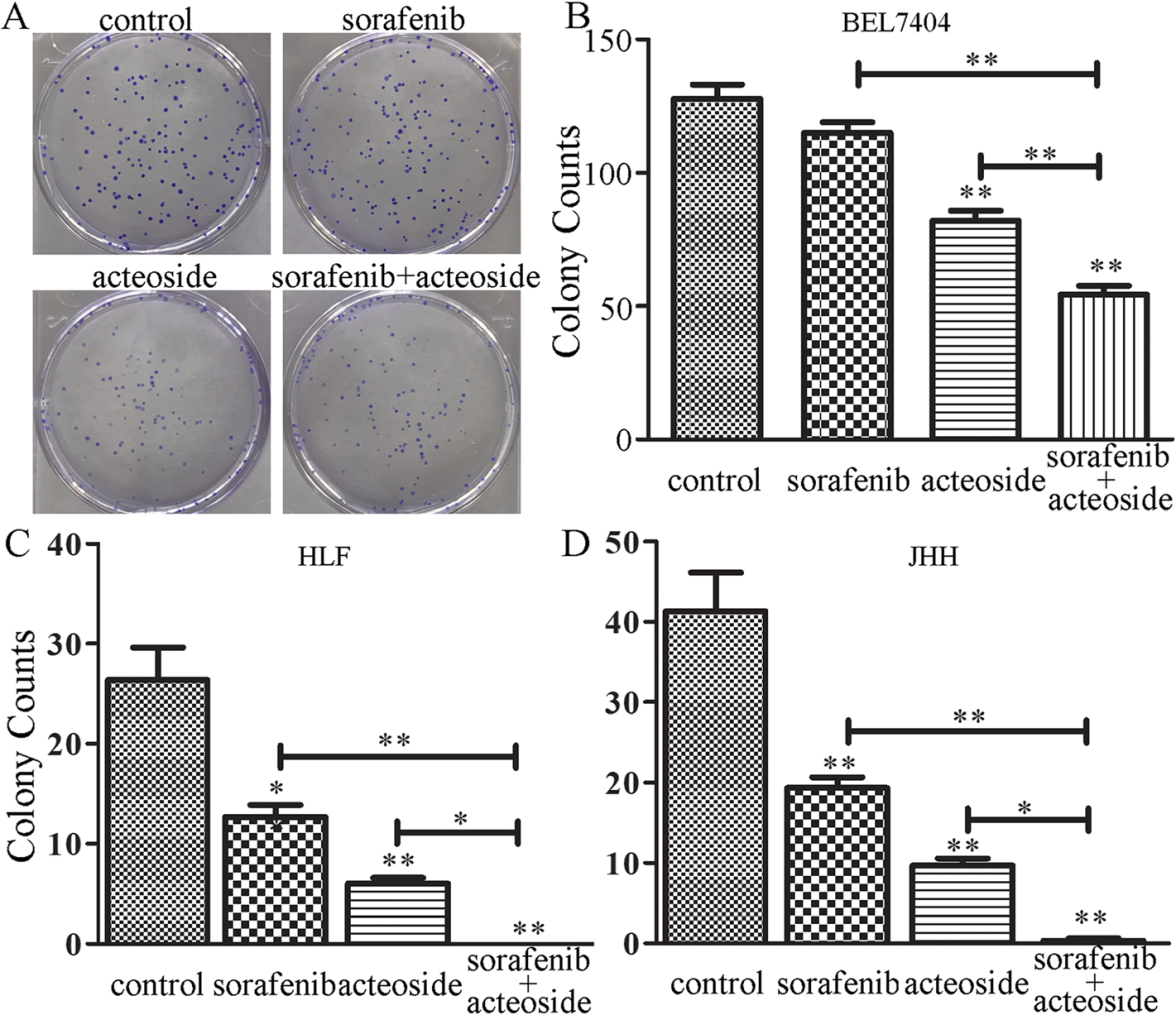
Effects of acteoside and sorafenib on colony formation of HCC cells. Cells were treated with acteoside (100 μM), sorafenib (2 μM) or their combination after seeded in 24-well plates (200 cells/well) overnight. Culture medium was replaced every 3 days. Colonies counting was performed after 12 days of culturing under a light microscope. Data are expressed as mean ± SD from three independent experiments. *,*p* < 0.05, **,*p* < 0.01

The three HCC cell lines (BEL7404, HLF and JHH7) were used for the cell proliferation assay. As shown in Fig. 1, the MTT assay revealed that A01 inhibited the proliferation of all the three HCC cells. To further ask whether the antiproliferative activity of A01 was due to its capability of inhibiting cell clonogenicity, the colony formation assay was performed. A01 at 100 μM showed a strong inhibition of colony formation in BEL7404 cells (Fig. 2a and b). When combined with sorafenib (2 μM), the inhibition potency was stronger than that of A01 or sorafenib alone. Similar results were also seen in the other two HCC cells, HLF and JHH-7 (Fig. 2c and d).

### Effects of A01 alone or in combination with sorafenib on cell migration of HCCs in vitro

**Fig. 3:**
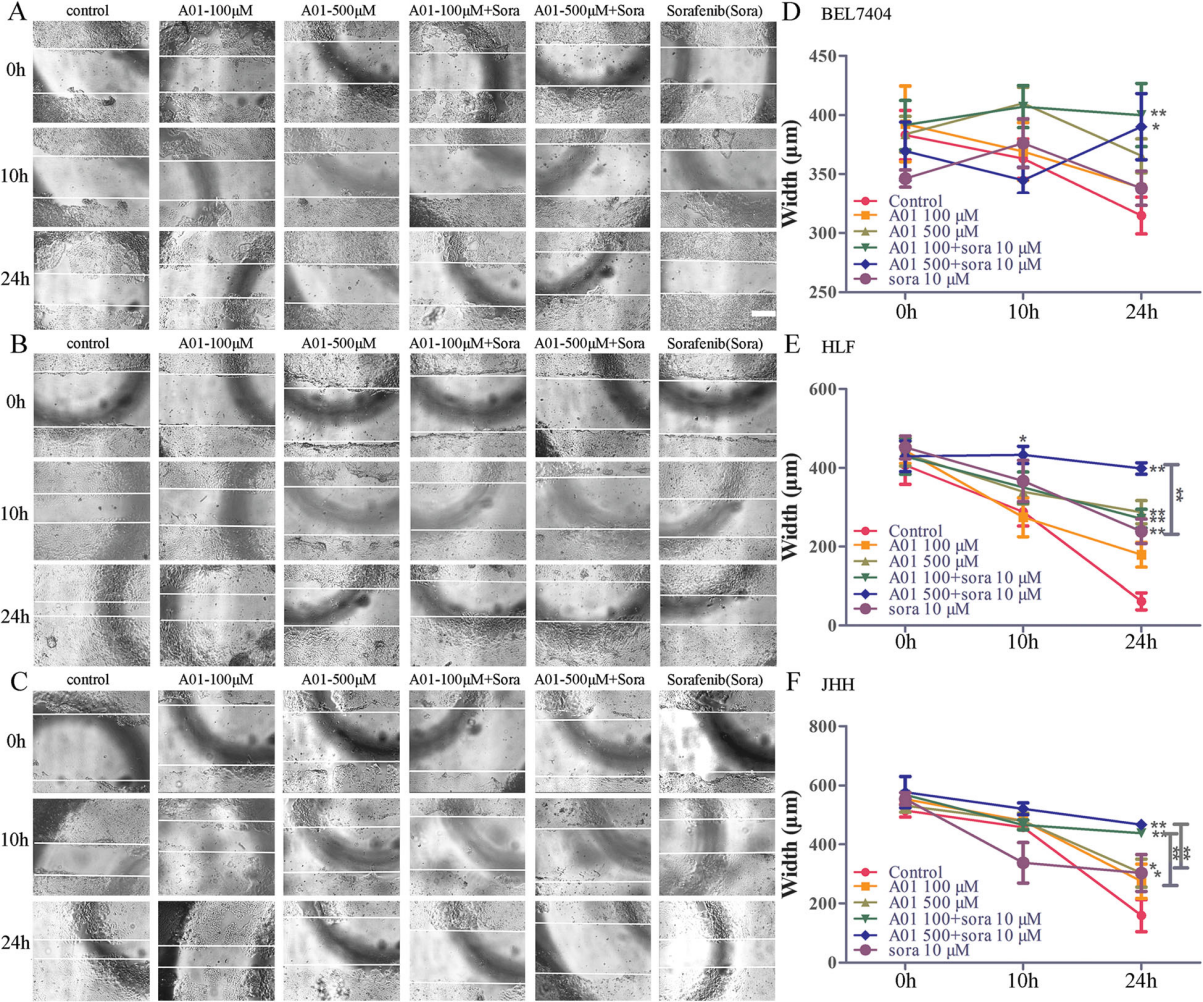
Wound healing assay in HCC cells. The cell layer was scratched with a pipette tip. Cells were then treated with acteoside (A01; 100 or 500 μM) and sorafenib (sora; 10 μM) alone or in combination. Cell migration was visualized at the indicated time point (0, 10 and 24 h) under an inverted light microscope at a magnification of × 100. Data are expressed as mean ± SD from three independent experiments. Scale bar, 500 μm. *,p < 0.05, **,*p* < 0.01 vs the control group or otherwise as specified

We next evaluated cell migration by using the scratch wound assay. As shown in Fig. 3, the combination of A01 (100 or 500 μM) and sorafenib (10 μM) significantly prevented wound healing in all the three HCC cells, when compared with the control group. For BEL7404 cells, both A01 (100 or 500 μM) and sorafenib (10 μM) only produced a tendency of prevention of wound healing with no statistical significance (Fig. 3a and d). For HLF cells, A01 at 500 μM and sorafenib at 10 μM inhibited wound healing. In particular, A01 (500 μM) combined with sorafenib (10 μM) produced a stronger inhibition of wound healing than sorafenib alone (Fig. 3b and e). For JHH cells, the wound healing was prevented by A01 (500 μM) and sorafenib (10 μM). The combination of A01 (100 μM) and sorafenib (10 μM) produced a stronger inhibition of wound healing than A01 (100 μM) or sorafenib (10 μM) alone. Moreover, A01 at 500 μM combined with sorafenib (10 μM) showed a stronger inhibition of wound healing than A01 (500 μM) or sorafenib (10 μM) alone (Fig. 3c and f).

### Effects of A01 alone or in combination with sorafenib on angiogenesis in vitro

**Fig. 4:**
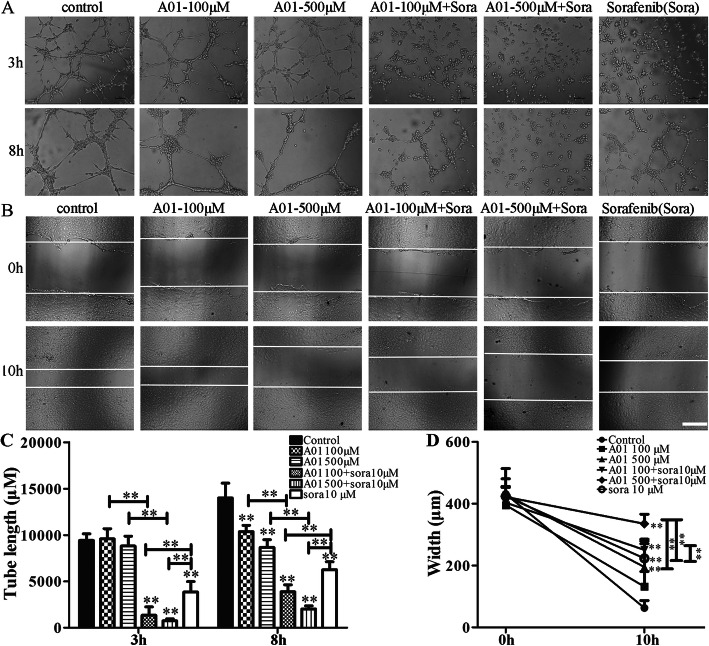
Effects of acteoside and sorafenib on angiogenesis in vitro. **a** and **c**, Matrigel tube-formation assay of HUVECs. Cells were treated with acteoside (A01; 100 or 500 μM) and sorafenib (sora; 10 μM) alone or in combination. Tube formation was then evaluated at 3 h and 8 h post-treatment. **b** and **d**, Wound healing assay in HUVECs. The cell layer was scratched with a pipette tip. Cells were then treated with acteoside (A01; 100 or 500 μM) and sorafenib (sora; 10 μM) alone or in combination. Cell migration was visualized at the indicated time point (0 and 10 h) under an inverted light microscope at a magnification of × 100. Scale bar, 500 μm. Data are expressed as mean ± SD from three independent experiments. **,*p* < 0.01 vs the control group or otherwise as specified

Angiogenesis is closely associated with tumor growth, progression and metastasis [16], and thus considered as one of the intervening targets for cancer treatment. We first performed tube formation assay to analyze the potential angiogenesis in vitro. As shown in Fig. 4, treatment for 3 h with sorafenib alone (10 μM) or the combination of A01 (100 and 500 μM) and sorafenib (10 μM) started to show inhibitory effects on the formation of vessel-like structures, i.e. the elongation and alignment of the HUVECs (Fig. 4a and c). Following a treatment for 8 h, A01 (100 and 500 μM), sorafenib (10 μM) as well as the combination of A01 and sorafenib all inhibited significantly the formation of vessel-like structures, with stronger inhibition by the combination of A01 and sorafenib than A01 or sorafenib alone (Fig. 4a and c). In addition, the cell migration of HUVECs is a key step to form new vessels during angiogenesis and is thereby investigated. As shown in Fig. 4b and d, A01 (500 μM), sorafenib (10 μM) as well as the combination of A01 (100 and 500 μM) and sorafenib (10 μM) all inhibited significantly cell migration of HUVECs. A01 (500 μM) combined with sorafenib (10 μM) produced stronger inhibition than A01 (500 μM) or sorafenib (10 μM) alone.

### Effects of A01 alone or in combination with sorafenib on the in vivo tumor growth of BEL7404 and JHH-7 HCC xenografts

**Fig. 5:**
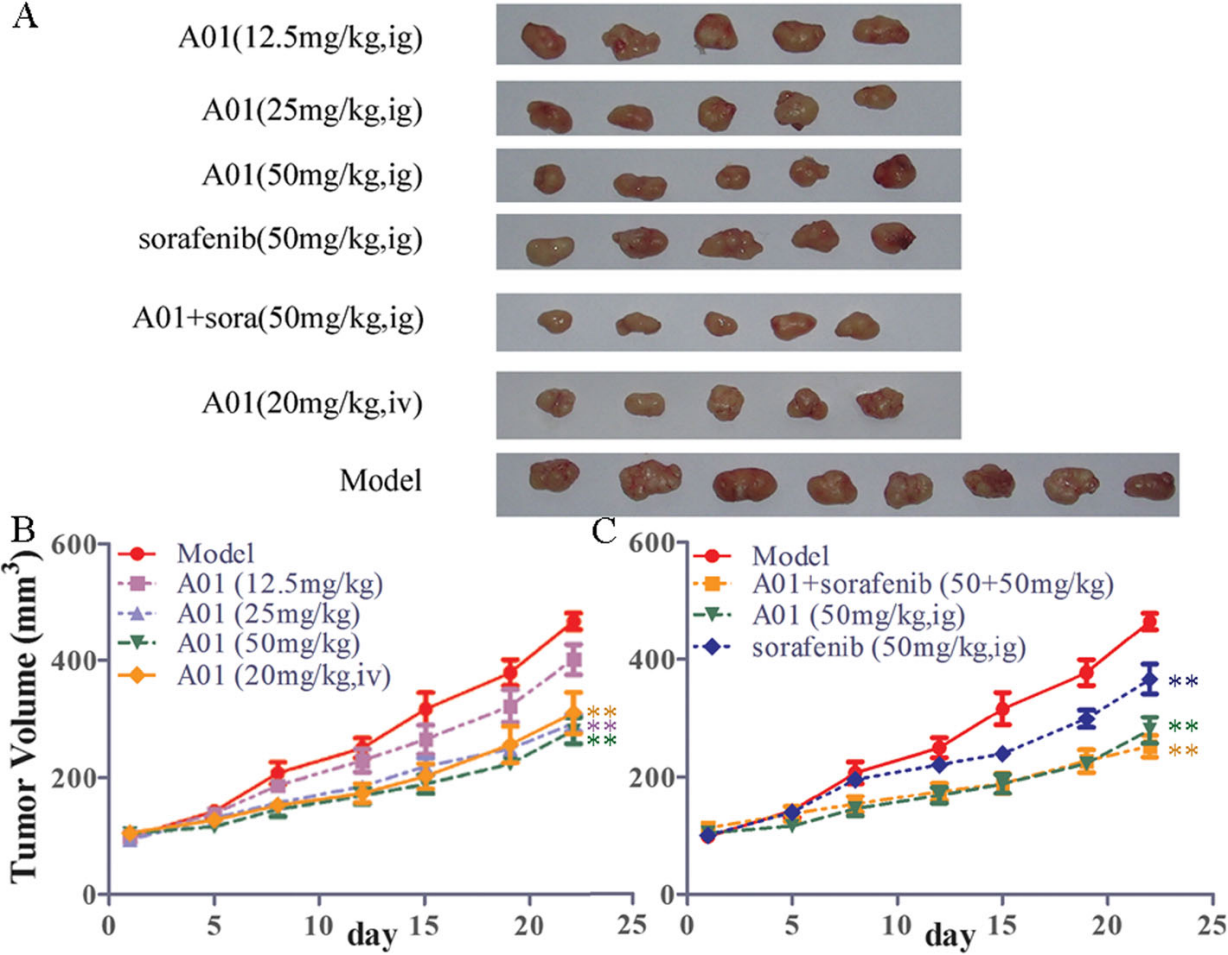
Effects of acteoside and sorafenib on the tumor growth of BEL7404 HCC xenografts in mice. The BEL7404 cell suspensions were prepared (3 × 10^6^ cells) and injected subcutaneously into the right flank of athymic nude mice. Fourteen days after the inoculation, tumor-bearing mice were treated via oral gavage with acteoside (A01; 12.5, 25 and 50 mg/kg), sorafenib alone (50 mg/kg) or in combination with A01 (50 mg/kg) once daily for 22 days. Tumor volume was measured twice a week. *N* = 5/group except for the model group (*n* = 8). Data are expressed as mean ± SEM. **,p < 0.01 vs the model group

**Fig. 6:**
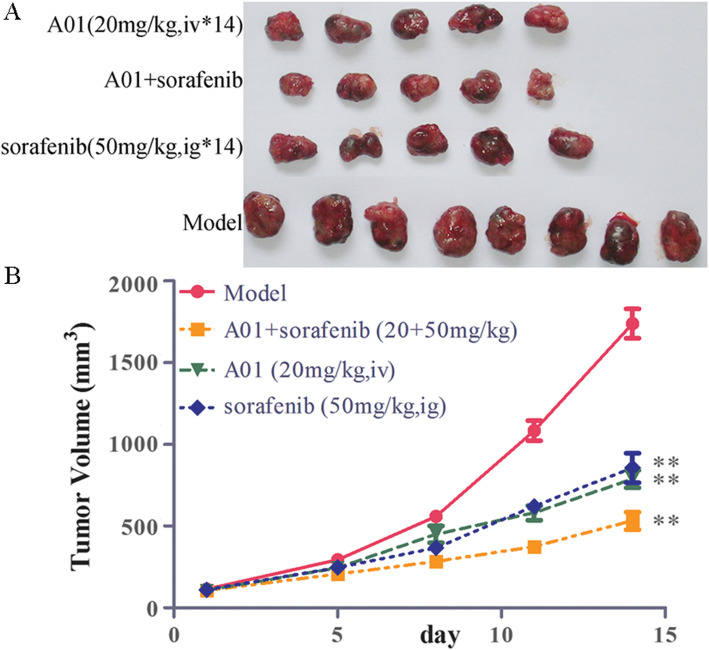
Effects of acteoside and sorafenib on the tumor growth of JHH-7 HCC xenografts in mice. The JHH-7 cell suspensions were prepared (3 × 10^6^ cells) and injected subcutaneously into the right flank of athymic nude mice. Fourteen days after the inoculation, tumor-bearing mice were treated with acteoside (A01; 20 mg/kg; iv), sorafenib alone (50 mg/kg;ig) or in combination with A01 (20 mg/kg; iv) once daily for 14 days. Tumor volume was measured twice a week. Data are expressed as mean ± SEM. N = 5/group except for the model group (n = 8). **,p < 0.01 vs the model group

To further evaluate the effectiveness of A01 alone or combined with sorafenib on the in vivo tumor growth of HCC, we established a subcutaneous xenograft nude mouse model using BEL7404 or JHH-7 cells. As shown in Fig. 5, the tumor volume was greatly decreased following treatment with A01 (25 and 50 mg/kg, via oral gavage; 20 mg/kg, via tail vein; all *p* < 0.01 vs model group) or sorafenib (50 mg/kg, via oral gavage; *p* < 0.01 vs model group) alone (Fig. 5a and b). The combination of A01 (50 mg/kg) and sorafenib (50 mg/kg) showed a stronger inhibition of tumor growth when compared with sorafenib alone (p < 0.01), but produced a similar effect as A01 alone (Fig. 5a and c). We next used the JHH-7 xenograft nude mice model to further test the possible anti-tumor efficacies of A01. As shown in Fig. 6, treatment with A01 (20 mg/kg, iv) or sorafenib (50 mg/kg, ig) produced a significant inhibition of tumor growth of JHH-7 xenograft (p < 0.01 vs model group), with greater inhibition when combined together (Fig. 6a and b; p < 0.01 vs A01 or sorafenib group alone).

### Possible modulation of kallikrein-related peptidase and p53 by acteoside

**Fig. 7:**
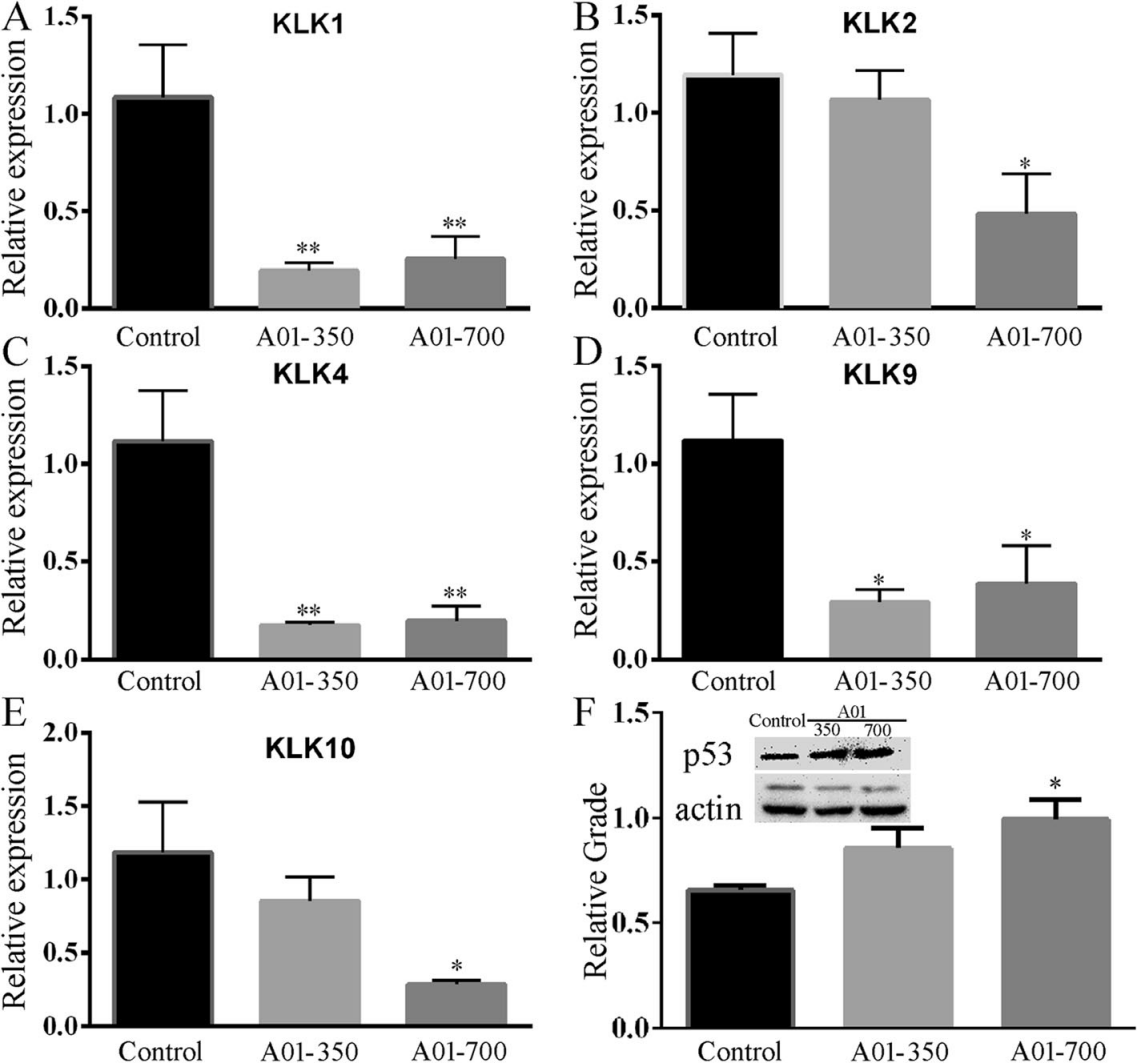
Changes of kallikrein-related peptidase (KLK) mRNA levels and tumor suppressor protein p53 levels following treatment of JHH-7 cells with acteoside. **a**-**e**, The down-regulation of KLK1, 2, 4, 9 and 10 mRNA levels was observed by acteoside (A01) at 350 or 700 μM (A01–350 or A01–700). **f**, Changes of p53 by western blot analyses. Treatment with acteoside (A01) increased p53 levels. Data are expressed as mean ± SEM (*n* = 4/group). *,*p* < 0.05 vs the control group; **,p < 0.01 vs the control group

The kallikrein-related peptidase (KLK) has been suggested as a cancer biomarker in the diagnosis and prognosis of various cancers, including hepatocellular carcinoma due to its dysregulated expression [17–19]. In this scenario, we detected gene expression of KLK1–15 in HCC JHH-7 cell line. As shown in Fig. 7, treatment with acteoside produced a significant inhibition of KLK1, 2, 4, 9 and 10 mRNA levels (Fig. 7a-e), with no significant changes of the rest of KLK1–15 (data not shown). In addition, we also detected the level of tumor suppressor protein p53 and revealed an increase by the treatment with acteoside (Fig. 7f).

## Discussion

The management of advanced HCC has long been intractable due to the inability of diagnosis at earlier stages and the chemoresistance of the tumor [5, 6]. Sorafenib, the multikinase inhibitor reported to inhibit cell proliferation and angiogenesis, together with another oral multikinase inhibitor lenvatinib has become the standard treatment of advanced HCC [3, 5, 20, 21]. However, the overall survival rates are modest in light of the low response rates and unsuitability in the clinical settings [3, 22]. A recent study showed that atezolizumab plus bevacizumab produced better clinical outcomes than sorafenib in patients with unresectable HCC, making this combination a possible new first-line treatment option for advanced HCC [7]. In this context, we have identified that the phenylethanoid glycoside, acteoside inhibited cell proliferation, colony formation and migration in the three human HCC cell lines BEL7404, HLF and JHH-7 as well as prohibited angiogenesis of HUVECs. The combination of acteoside and sorafenib produced stronger inhibition of cell colony formation and migration of the HCC cells as well as of angiogenesis of HUVECs. Acteoside presented the antitumor efficacy in BEL7404 or JHH-7 xenograft nude mice model, with an enhancement when combined with sorafenib in inhibiting the growth of JHH-7 xenograft. Further analyses revealed an increase in p53 as well as a decrease of some KLK genes. Accordingly, the possible use of acteoside as adjuncts in the treatment of advanced HCC in the clinic should be considered.

Mounting evidence has shown the antitumor effects of acteoside in various tumor cells such as B16 melanoma cells, glioblastoma cells, colorectal cancer cells, oral squamous cell carcinoma cells, prostate cancer cells and human breast adenocarcinoma cells [11–13, 23–25] or in animals bearing tumors [12]. Despite that, few studies have focused on the effects of acteoside on HCC in vitro or in vivo except one study using diethylnitrosamine as the carcinogen to induce hepatocarcinogenesis in rats and revealing the chemoprevention by acteoside [26]. In the current study, we showed the inhibition by acteoside of cell proliferation, colony formation and migration in HCC cell lines as well as tumor growth in mice bearing HCC cell line xenografts, thereby directly demonstrating the antitumor effects of acteoside in HCC. Furthermore, the combination of acteoside and sorafenib produced even stronger antitumor effects. Combined with the evidence that acteoside is protective against liver injuries [27–29] with no toxic effects in animals and no mutagenicity [30], it seems to be feasible for the future application of the combination therapy of acteoside and sorafenib in patients with advanced HCC in the clinic.

The human kallikrein family comprises 15 homologous, single-chain, secreted trypsin- or chymotrypsin-like serine proteases (KLK1–15) and has been implicated in regulation of tumor growth, neoplastic progression, angiogenesis and metastasis [18, 31]. In particular, the KLKs have been suggested as a cancer biomarker in the diagnosis and prognosis of various cancers, including hepatocellular carcinoma due to its dysregulated expression [17–19]. For example, KLK1 is proposed as the biomarker of gastric cancer due to the elevation of KLK1 levels in gastric neoplasm and the prevention of tumor growth in gastric cancer by inhibition of KLK1 activity with kallikrein-binding protein (KBP) [32]. In this scenario, we observed a significant inhibition of KLK mRNA levels by acteoside (KLK1, 2, 4, 9 and 10). Application of KBP, which specifically binds to tissue kallikrein and inhibits kallikrein activity, was reported to suppress growth of HCC in mice possibly via its anti-angiogenic activity [33], suggesting the possible involvement of KLKs in the progression of HCC. In addition, the tumor suppressor protein p53 is able to induce senescence, apoptosis as well as cell cycle arrest. Increased p53 expression confers enhanced chemosensitivity in cancer cells [34]. In this regard, our western results showed an increase in p53 levels following treatment with acteoside. Taken together, it is reasonable to infer that acteoside exerts the antitumor effects on HCC possibly through its up-regulation of p53 as well as inhibition of KLK and angiogenesis.

It should be noted that the direct molecular target to which acteoside could directly bind is still unclear. Our preliminary data using molecular docking revealed a direct binding of acteoside with kallikrein (data not shown). Further experiments would be necessary to clarify this presumption. In addition, as for the doses of acteoside used for cell assay in the study 10–50 times higher than that of sorafenib (10 μM), firstly we chose those doses of acteoside in accordance with the dose-response relationship in the MTT assay. Second, the doses we used (100, 500 μM) were comparable to previous studies [35], in which acteoside (100 μM) inhibited cell proliferation of colorectal cancer cell. Last, despite the higher dose in the cell assay, acteoside produced a pronounced inhibition of tumor growth in the HCC xenograft nude mice model in our study (25 and 50 mg/kg), effects being similar to sorafenib (50 mg/kg). For sorafenib, it was used in our study at the dose of 10 μM at the cellular level. This dose is comparable to previous studies in which sorafenib was found to inhibit the cell viability with an IC_50_ of 4.0–6.5 μM in the HCC cell lines of PLC/PRF/5 and HepG2 cells. In contrast, the dose of sorafenib used to suppress the growth of HCC xenografts in SCID female mice was 30 mg/kg (p.o. by gavage) [36]. In the clinic, the recommended dosage of sorafenib for patient is 400 mg per time (2*0.2 g), twice a day (800 mg per day in total). This dosage for sorafenib used in the clinic is in contrast to that used in cell assay (IC50 value in μM level), possibly due to its poor absorbability under in vivo conditions. For acteoside, based on its dosage in mice (50 mg/kg, i.g.), its equivalent dose for human (60 kg bw) is about 250 mg. This dosage is comparable to that of sorafenib used in the clinic and should be acceptable for the patients.

## Conclusions

In conclusion, acteoside is capable of exerting an antitumor effect in HCC cell lines and in nude mice bearing HCC cell line xenografts, effects possibly via increasing p53 levels as well as prohibiting KLK and angiogenesis. Acteoside could be useful as an adjunct in the treatment of advanced HCC in the clinic.

## Supplementary information

**Additional file 1: Supplemental Figure 1.** Full image of western blotting bands for p53 and its internal control actin shown in Fig. 7f. Membrane was pre-cut according to the molecular weight of p53 and Bax. After p53 (or Bax) staining, the membrane was stripped with stripping buffer and reprobed with the actin antibody (or Bcl-2). Note that the remanant obscure p53 band was seen right above the actin band.

**Additional file 2.**

## Data Availability

All analyzed data are included in this published article. The original data are available upon request to the corresponding author.

## Abbreviations

A01: acteoside
HCC: Hepatocellular carcinoma
HUVEC: Human umbilical vein endothelial cell
KLK: kallikrein-related peptidase
MTT: 3-(4,5-Dimethylthiazol-2-yl)-2,5-diphenyltetrazolium bromide
RIPA: radio-immunoprecipitation assay buffer
