# Supplementary material for: Acteoside as a potential therapeutic option for primary hepatocellular carcinoma: a preclinical study
Source: BMC Cancer. 2020 Sep 29;20:936. doi: 10.1186/s12885-020-07447-3 (PMC7526186; doi:10.1186/s12885-020-07447-3)
Supplement: Supplementary file 1 — Additional file 1: Supplemental Figure 1. Full image of western blotting bands for p53 and its internal control actin shown in Fig. 7f. Membrane was pre-cut according to the molecular weight of p53 and Bax. After p53 (or Bax) staining, the membrane was stripped with stripping buffer and reprobed with the actin antibody (or Bcl-2). Note that the remanant obscure p53 band was seen right above the actin band. [file 12885_2020_7447_MOESM1_ESM.docx]

P53

Bax


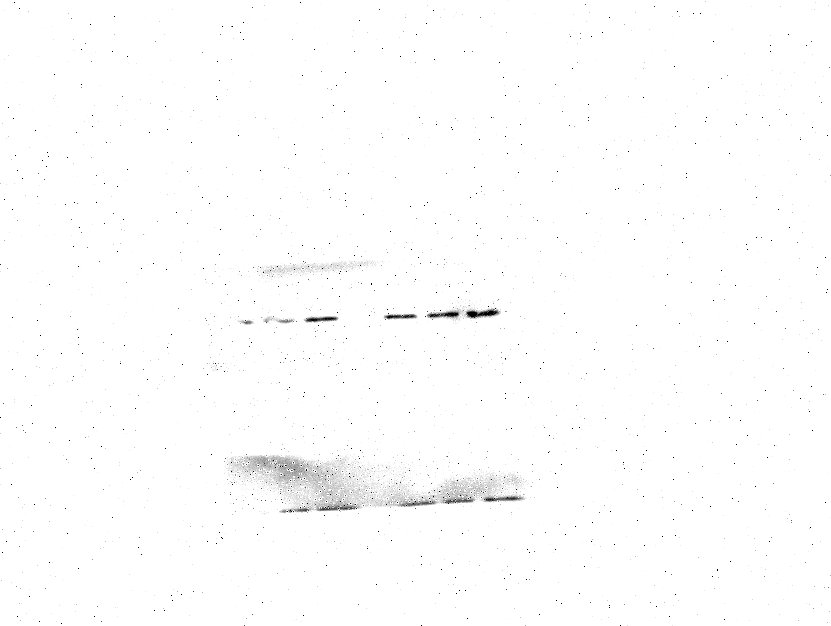


Actin (after p53 was stripped with stripping buffer)

Bcl-2


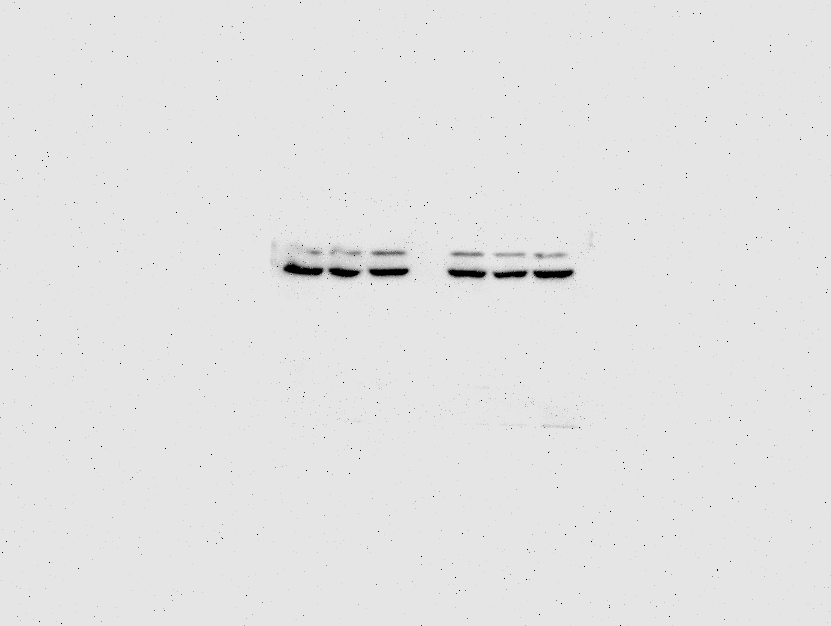


**Supplemental Fig.1** Full image of western blotting bands for p53 and its internal control actin shown in Figure 7F. Membrane was pre-cut according to the molecular weight of p53 and Bax. After p53 (or Bax) staining, the membrane was stripped with stripping buffer and reprobed with the actin antibody (or Bcl-2).

Note that the remanant obscure p53 band was seen right above the actin band.
