# Supplementary material for: Acteoside as a potential therapeutic option for primary hepatocellular carcinoma: a preclinical study
Source: BMC Cancer. 2020 Sep 29;20:936. doi: 10.1186/s12885-020-07447-3 (PMC7526186; doi:10.1186/s12885-020-07447-3)

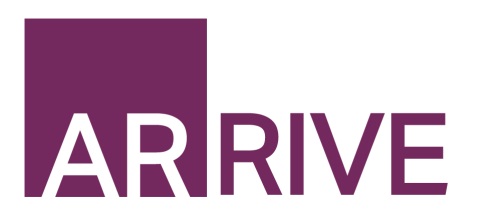


The ARRIVE Guidelines Checklist

Animal Research: Reporting In Vivo Experiments

Carol Kilkenny^1^, William J Browne^2^, Innes C Cuthill^3^, Michael Emerson^4^ and Douglas G Altman^5^

*^1^The National Centre for the Replacement, Refinement and Reduction of Animals in Research, London, UK, ^2^School of Veterinary Science, University of Bristol, Bristol, UK, ^3^School of Biological Sciences, University of Bristol, Bristol, UK, ^4^National Heart and Lung Institute, Imperial College London, UK, ^5^Centre for Statistics in Medicine, University of Oxford, Oxford, UK.*

|  | | ITEM | RECOMMENDATION | Section/ Paragraph |
| --- | --- | --- | --- | --- |
| 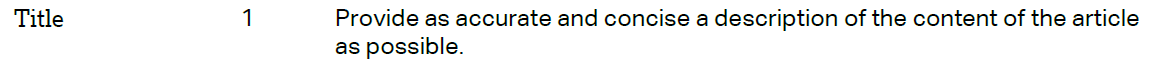 | | | Title, Paragraph 1 |  |
| 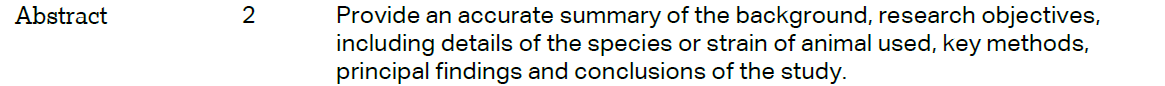 | | | Abstract, Paragraphs 1-4 |  |
| INTRODUCTION | | |  |  |
| 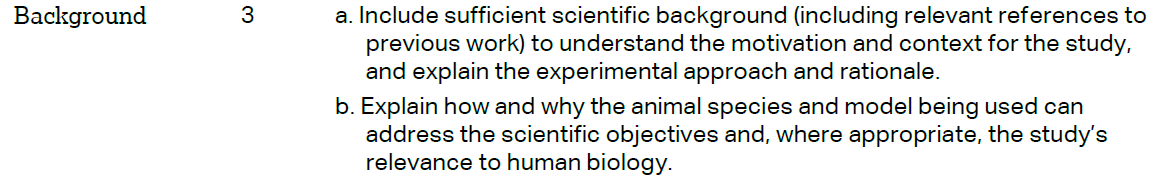 | | | Background, Paragraphs 1-3 |  |
| 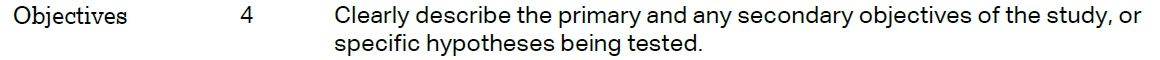 | | | Background, Paragraphs 1-2 |  |
| METHODS | | |  |  |
| 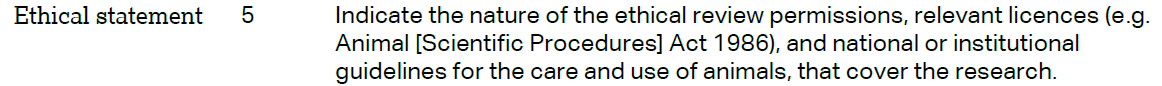 | | | Methods, Paragraph 3 |  |
| 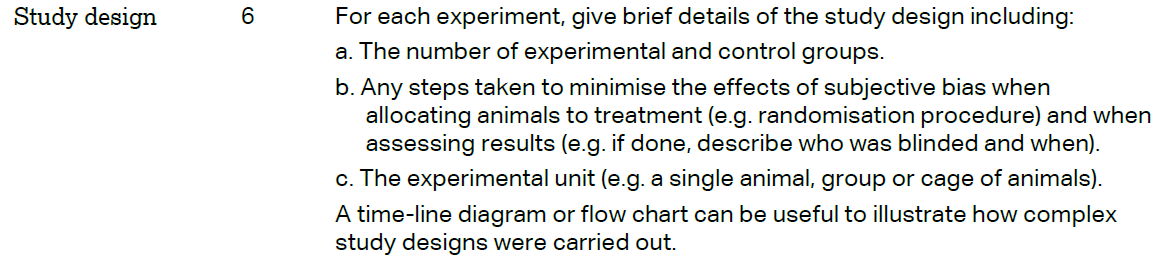 | | | Methods, Paragraph 10;  Figures 5 and 6 with the legends. |  |
| 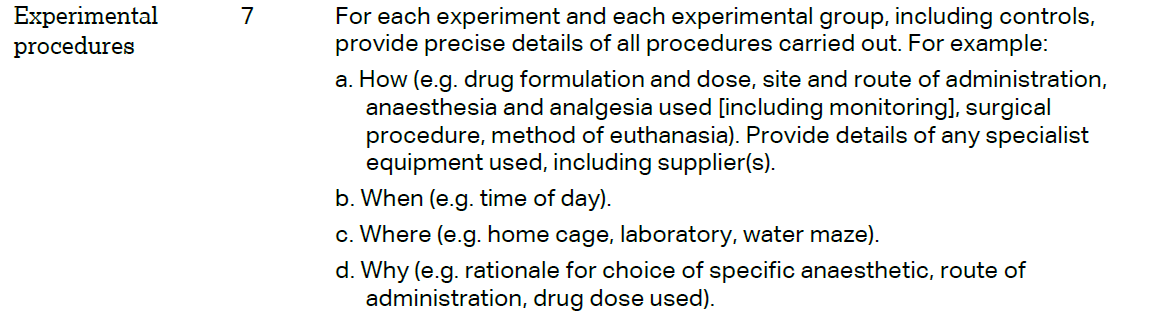 | | | Methods, Paragraph 10. |  |
| 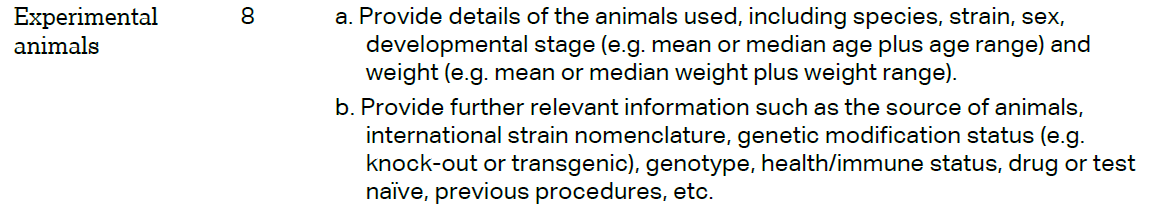 | | | Methods, Paragraph 3. |  |

The ARRIVE guidelines. Originally published in *PLoS Biology*, June 2010^1^

| 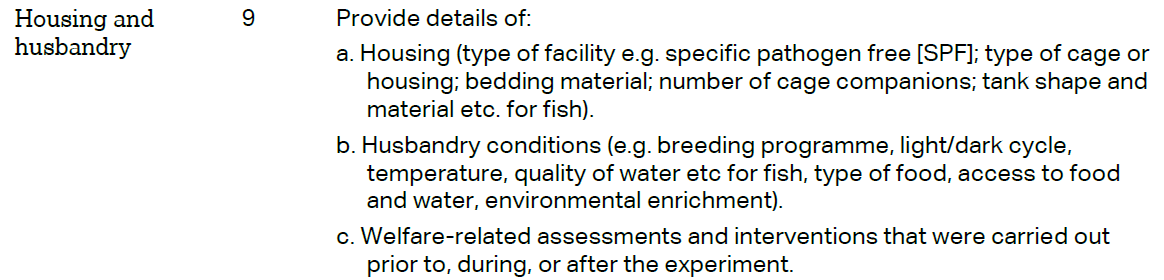 | Methods, Paragraph 3 | |
| --- | --- | --- |
| 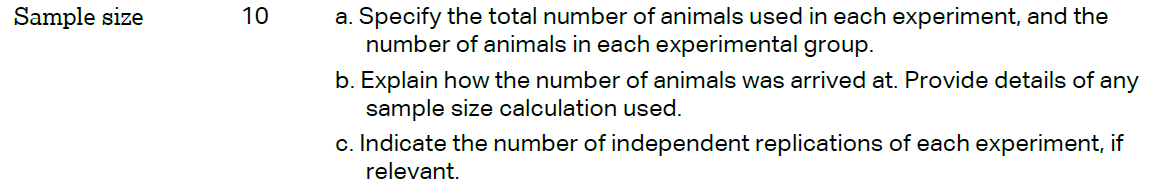 | Methods, Paragraph 10; | |
| 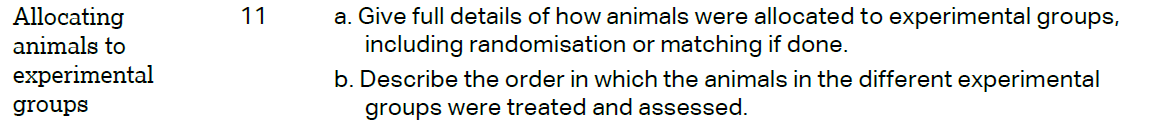 | Methods, Paragraph 10; | |
| 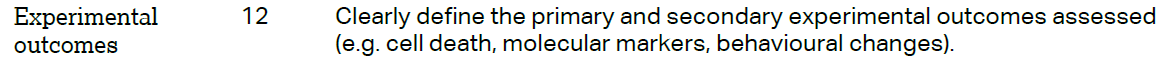 | Methods, Paragraph 10; | |
| 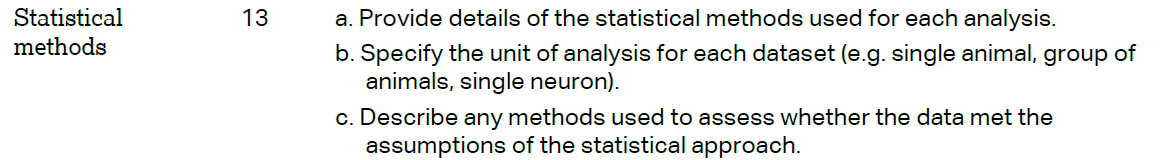 | Methods, Paragraph 11 | |
| RESULTS |  | |
| 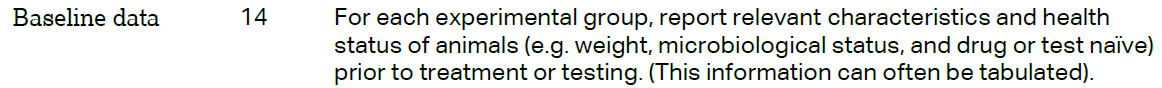 | Methods, Paragraph 10; | |
| 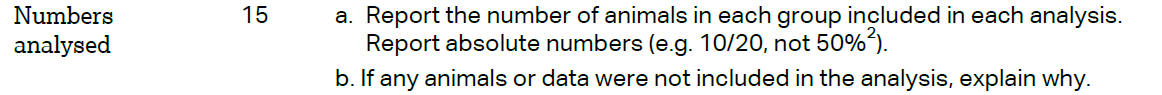 | Figures 5 and 6 and the legends | |
| 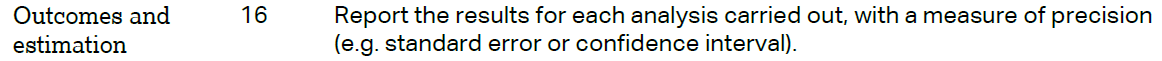 | Results, paragraph 4; Figures 5 and 6 and the legends | |
| 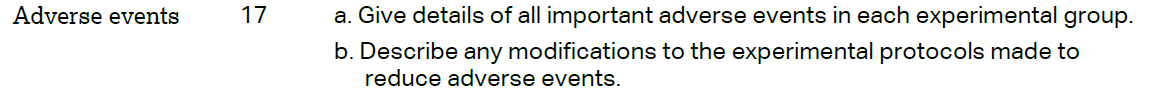 | N/A | |
| DISCUSSION |  | |
| 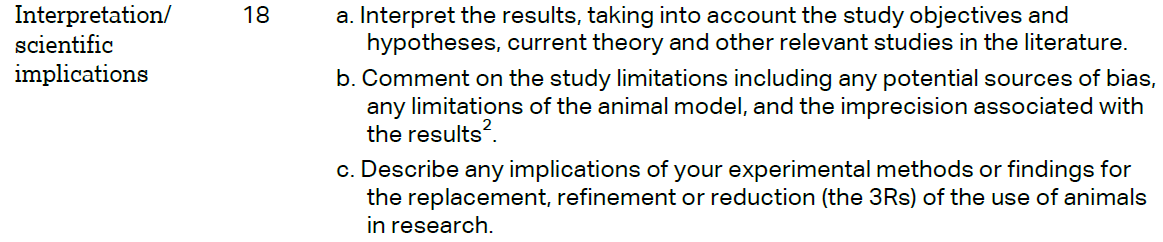 | Discussion, paragraphs 1, 2 and 4 | |
| 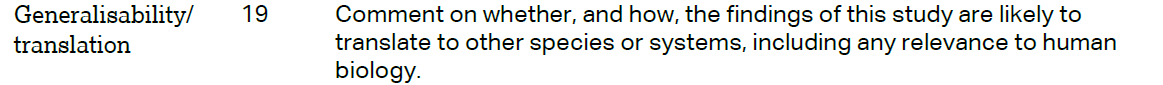 | Discussion, paragraphs 1 and 2. | |
| 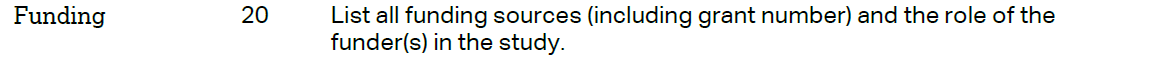 | | Declarations, paragraph 5 |


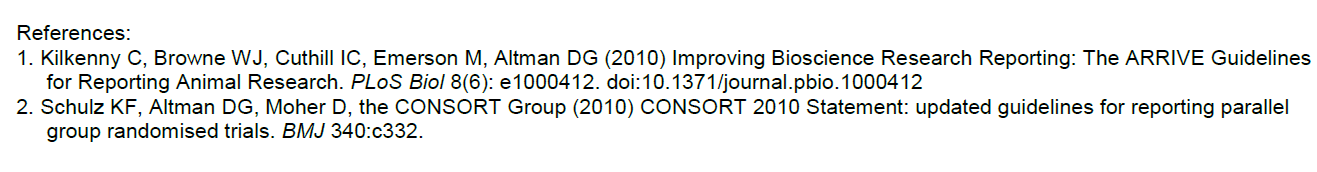

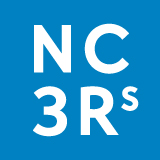

Supplement: Supplementary file 2 — Additional file 2. [file 12885_2020_7447_MOESM2_ESM.docx]
